# Ratiometric fluorescent probe for enantioselective detection of D-cysteine in aqueous solution

**DOI:** 10.3762/bjoc.7.176

**Published:** 2011-11-09

**Authors:** Xiao-bo Zhou, Wing-Hong Chan, Albert W M Lee, Chi-Chung Yeung

**Affiliations:** 1 Department of Chemistry, Hong Kong Baptist University, Kowloon Tong, Hong Kong SAR, China

**Keywords:** chemosensor, D-cysteine, enantioselectivity, fluorescence, ratiometric

## Abstract

A ratiometric fluorescent probe based on a Cd^2+^–**ACAQ** complex was designed and demonstrated for the chemo- and enantioselective detection of cysteine in 99:1 buffered HEPES:ACN solutions. Under the measuring conditions, the sensor demonstrates high selectivity toward Cys against Hcy and GSH, and an enantioselectivity of 3.35 can be achieved for antipodal forms of Cys.

## Introduction

**Figure 1:**
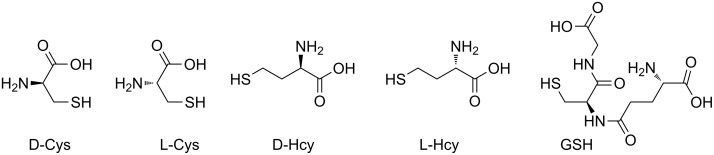
Structures of biological active thiols.

The rapid, sensitive, and selective sensing of biologically relevant thiols including cysteine (Cys), homocysteine (Hcy) and glutathione (GSH) has attracted significant interest in the sensor community (Figure 1) [1]. All three of these thiols are present in living cells at different concentration levels and play vital roles in controlling redox-related biological processes [2]. Abnormal levels of Cys and Hcy are associated with a variety of human diseases [3–5]. Hence, the development of efficient and selective methods capable of quantifying biological thiols under physiological conditions is of interest in a wide variety of disciplines including chemistry and medicine. Owing to the intrinsic sensitivity of fluorescence, the development of fluorescent probes for thiols has been extensively invesigated. The subject has been recently reviewed by Yoon and coworkers [6]. A number of novel sensing strategies have been designed that mostly relied on the high nucleophilicity of the sulfhydryl group. For instance, Michael addition of thiols onto fluorescent “acceptors” can trigger a change in the fluorescence outputs of the sensory materials [7–11]. The facile cyclization of β- or γ-aminothiols with aldehydes containing fluorescent probes forms the basis for the development of several fluorescent probes [12–17]. In addition, cleavage of disulfide-based probes by thiols [18–21] and other thiol-sensing tactics have been documented [22–28]. As an improved sensing device, a ratiometric fluorescence probe monitors the emission intensities at two different wavelengths, which should provide a built-in correction for instrumental defects and photodegradation of sensing materials. In this context, a few ratiometric ﬂuorescent chemosensors for the detection of thiols have been constructed [29–31]. Recently, on the basis of a native chemical-ligation reaction, Lin and coworkers reported a FRET-based probe suitable for ratiometric imaging of cysteine in living cells [31]. Therefore, it is a challenging task to design probes for the detection of Cys. In addition, to our knowledge, an enantioselective fluorescent chemosensor for D-Cys has not been reported in the literature. It is well known that D-Cys is a powerful inhibitor of *E. coli*, some strains of which can have very serious effects on human health [32].

On the basis of multipoint electrostatic interactions and structure complementarity of the host–guest, we have rationally designed and synthesised a bis(spiropyran) as a fluorescence turn-on probe for selective binding of GSH [33]. We have also developed the first spiropyran–metal sensing ensemble for selective detection of Cys and Hcy [34]. Continuing our interests in the design and the synthesis of fluorescent chemosensors for biologically active molecules, we describe herein a ratiometric fluorescent probe that can be exploited for the enantioselective recognition of cysteine in aqueous solutions. Interestingly, the probe is only slightly responsive to Hcy and GSH.

## Results and Discussion

**Figure 2:**
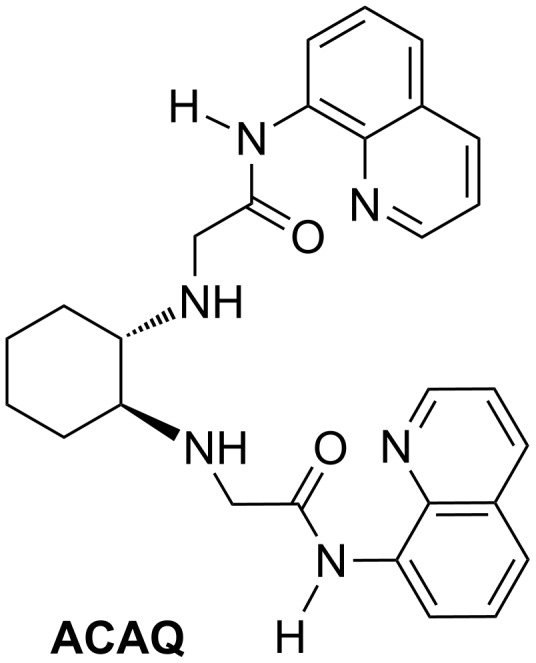
The structure of **ACAQ**.

N-Alkylation of *trans*-1,2-di**a**mino**c**yclohexane with a pair of 9-carbox**a**mido**q**uinoline moieties through methylene linkers afforded multifunctionalized **ACAQ** (Figure 2). Bearing six metal ligating sites, **ACAQ** was developed by us as a cell-permeable, fluorescent, ratiometric sensing probe for the detection of Zn^2+^ and Cd^2+^ [35].

**Figure 3:**
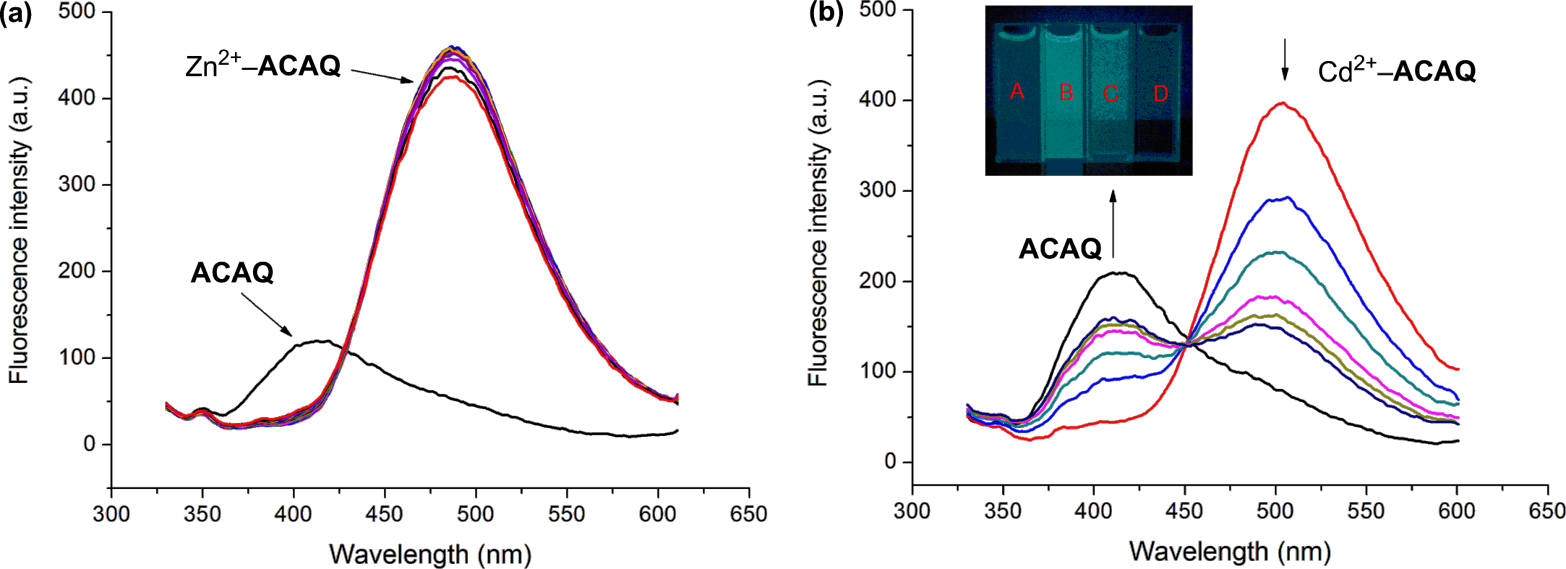
Emission spectra of (a) Zn^2+^–**ACAQ** or (b) Cd^2+^–**ACAQ** complex (5 μM) at increasing concentrations of Cys (0, 1.0, 2.0, 3.0, 4.0, 5.0 equiv) in buffered solution (10 mM, HEPES, 80% ACN, pH 7.4) at 298 K. Excitation wavelength was 315 nm. Inset in (b): Visible emission (irradiated by 365 nm light) observed. A: Only **ACAQ**; B: Cd^2+^–**ACAQ**; C: Cd^2+^–**ACAQ/**0.5 equiv Cys; D: Cd^2+^–**ACAQ/**2.0 equiv Cys.

Operating according to the intramolecular charge-transfer (ICT) mechanism, complexation of **ACAQ** with either Cd^2+^ or Zn^2+^ triggers a ratiometric change in the emission band of **ACAQ**. A redshift of the emission band of **ACAQ** from 400 nm to 500 nm was observed as a result of the metal–host interaction. Adding a quantity of cysteine of up to 25 µM into a 2:8 aqueous acetonitrile buffered solution (HEPES, pH 7.40) of the Zn^2+^–**ACAQ** complex did not induce any change in its fluorescence (Figure 3a). Conceivably, the relatively weak interaction between zinc and cysteine did not perturb the structure of the Zn^2+^–**ACAQ** complex, as was evident by the intact nature of its fluorescence spectrum. On the other hand, progressive addition of cysteine to the buffered solution of the corresponding cadmium complex (Cd^2+^**–ACAQ**) can induce a distinctive ratiometric fluorescence change characterized by the formation of an obvious isoemissive point at 450 nm (Figure 3b). We envisioned that the strong binding affinity of cysteine to Cd^2+^ perturbs its interaction with **ACAQ** in such a way that the ICT process within the complex is greatly retarded. When 5 equivalents of Cys is introduced into the probe solution, the emission spectrum of the resultant solution is very similar to that of the original spectrum of pure **ACAQ**.

To define the optimium operative conditions of the sensing probe for detecting cysteine, different aqueous solvent systems were chosen for subsequent investigations. On the basis of the results of the fluorescence titrations, the respective binding constants under different measuring conditions were estimated from the nonlinear fits (Table 1). In 20% aqueous buffer solutions, acetonitrile (ACN) emerged as the best cosolvent, among other polar solvents, to favor the formation of the most stable complex between the probe and Cys, as characterized by the highest association constant. When a higher content of water was used, the association constant of Cd^2+^–**ACAQ** and L-cysteine decreased.

**Table 1:** The association constants K_ass_ of Cd^2+^–**ACAQ** (5 µM) and L-cysteine in different solvents.

solvent system^a^	K_ass_ (M^−1^)	R^2^

DMSO:HEPES = 8:2	(6.42 ± 1.56) × 10^4^	0.9962
MeOH:HEPES = 8:2	(0.39 ± 0.24) × 10^4^	0.9715
EtOH:HEPES = 8:2	(2.50 ± 0.68) × 10^4^	0.9860
ACN:HEPES = 8:2	(1.20 ± 0.30) × 10^5^	0.9790
ACN:HEPES = 5:5	(0.42 ± 0.12) × 10^4^	0.9923
ACN:HEPES = 2:8	(0.66 ± 0.03) × 10^4^	0.9977

^a^HEPES in 10 mM at pH 7.4.

**Figure 4:**
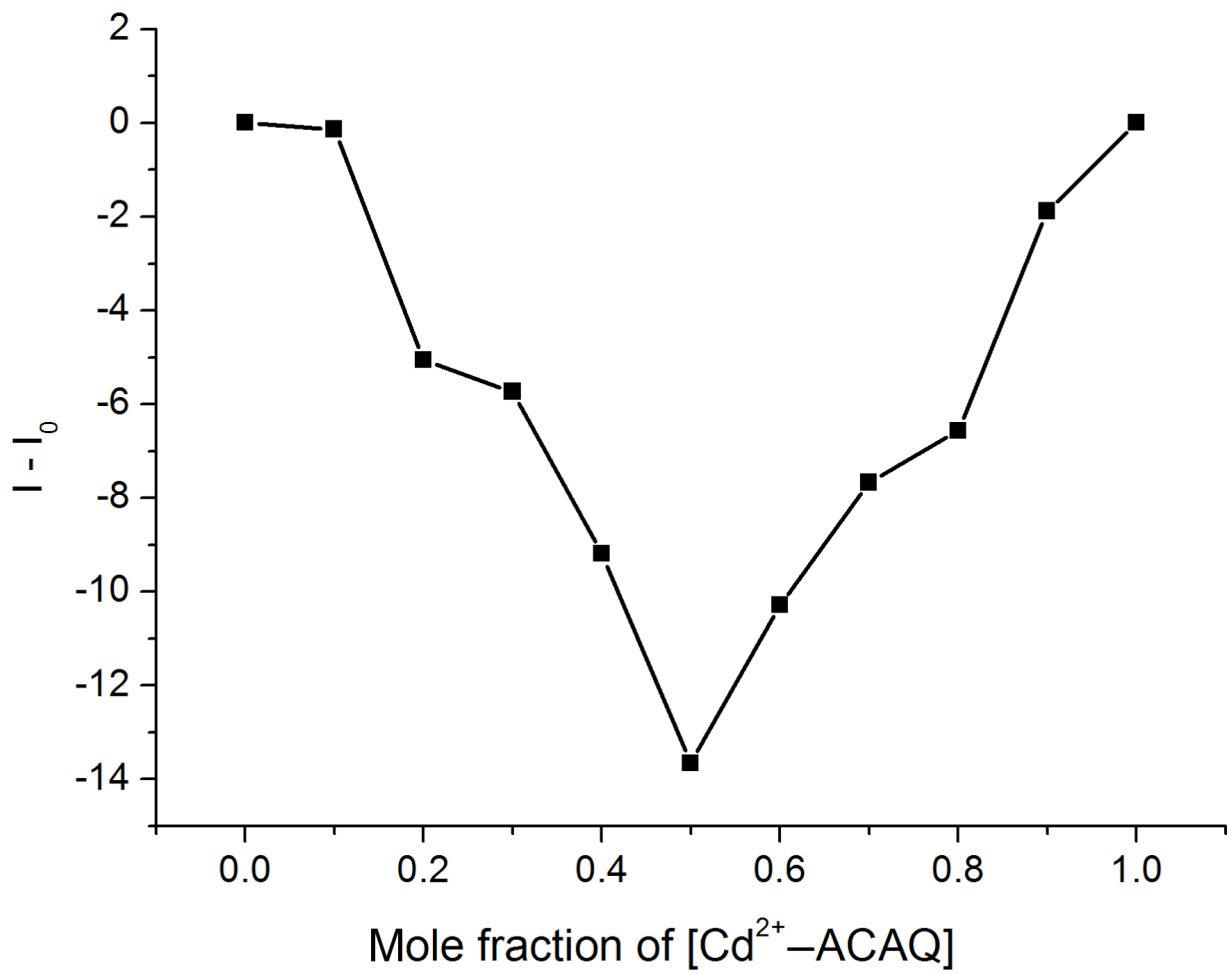
Job’s plot of Cd^2+^**–ACAQ** in 10 mM HEPES buffer (pH 7.4) at 298 K. The sum of the concentrations of the host and D**-**Cys is 5 µM (emission at 500 nm).

It is noteworthy that irrespective of the exact solvent composition of the aqueous solutions the binding affinity of the probe to Cys is fairly strong, as evidenced by the binding constants. On the other hand, the Job’s plot confirms the 1:1 binding stoichiometry of the sensing ensemble and Cys (Figure 4).

**Figure 5:**
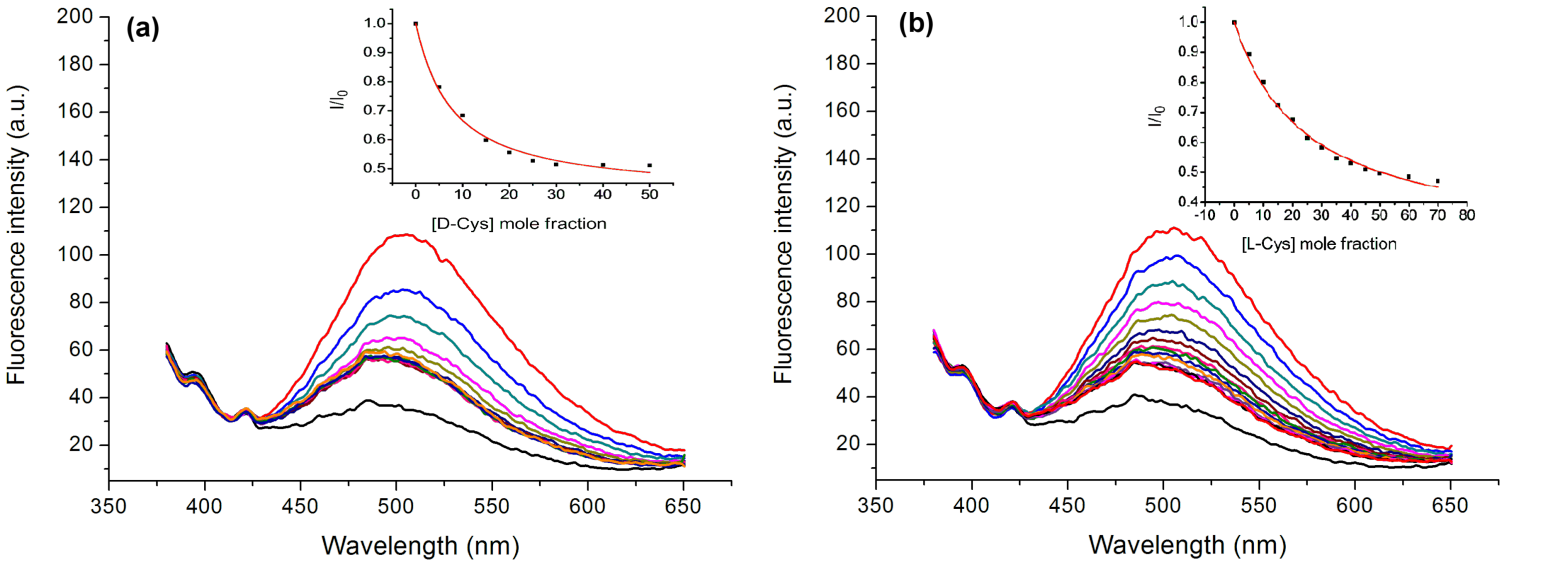
Fluorescence spectra of Cd^2+^**–ACAQ** (10 µm) upon the titration of (a) D-Cys and (b) L-Cys in buffer solution (10 µM, HEPES, 1% ACN, pH 7.4 ) at 298 K with λ_ex_ = 350 nm. Inset is the titration data point and the nonlinear least-squares fitting curve at 500 nm.

To explore the chiral recognition ability of the sensory ensemble, a comparative study of the binding of the sensor to antipodal forms of Cys in different media was undertaken. On the basis of fluorescence titration results, as shown in Table 2, the sensory probe displays a modest enantioselectivity of 3.35 conferred by the chiral *trans*-1,2-diaminocyclohexane moiety for recognizing D-cysteine in 1% ACN/HEPES buffered solutions (pH 7.4) (Figure 5). Apparently, owing to the structure complementarity of the host–guest, D-cysteine outperforms its enantiomer in forming a more stable complex with Cd^2+^–**ACAQ**.

**Table 2:** Association constants K_ass_ (M^–1^) of Cd^2+^–**ACAQ**^a^ with antipodal Cys in different solvents.

ACN:HEPES^b^	guest	K_ass_ (M^–1^)	enantioselectivity

8:2	D-Cys	(2.07 ± 0.37) × 10^5^	K_D_/K_L_ = 1.73
	L-Cys	(1.20 ± 0.30) × 10^5^	
5:5	D-Cys	(1.54 ± 0.09) × 10^4^	K_D_/K_L_ = 3.67
	L-Cys	(0.42 ± 0.12) × 10^4^	
1:99^c^	D-Cys	(2.78 ± 0.36) × 10^4^	K_D_/K_L_ = 3.35
	L-Cys	(0.83 ± 0.07) × 10^4^	

^a^In 5 µM; ^b^HEPES in 10 mM at pH 7.4; ^c^in 10 µM.

**Figure 6:**
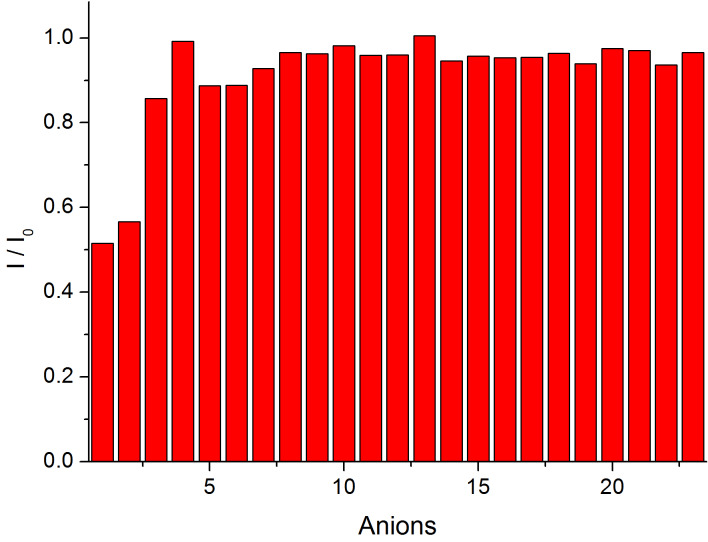
Fluoresence emission change of Cd^2+^–**ACAQ** at 500 nm in response to the addition of 15 equiv amino acids in 1:99 ACN:HEPES buffer solution (pH 7.4) at 298 K (excitation at 350 nm): 1. D-Cys; 2. L-Cys; 3. GSH; 4. L-Met; 5. DL-Hcy; 6. *N*-Ac-L-Cys; 7. MPA; 8. L-Asp; 9. D-Asp; 10. D-Glu; 11. L-Glu; 12. D-Lys; 13. L-Lys; 14. D-Ser; 15. L-Ser; 16. D-Tyr; 17. L-Tyr; 18. D-Val; 19. L-Val; 20. D-Phe; 21. L-Phe; 22. D-His; 23. L-His.

**Figure 7:**
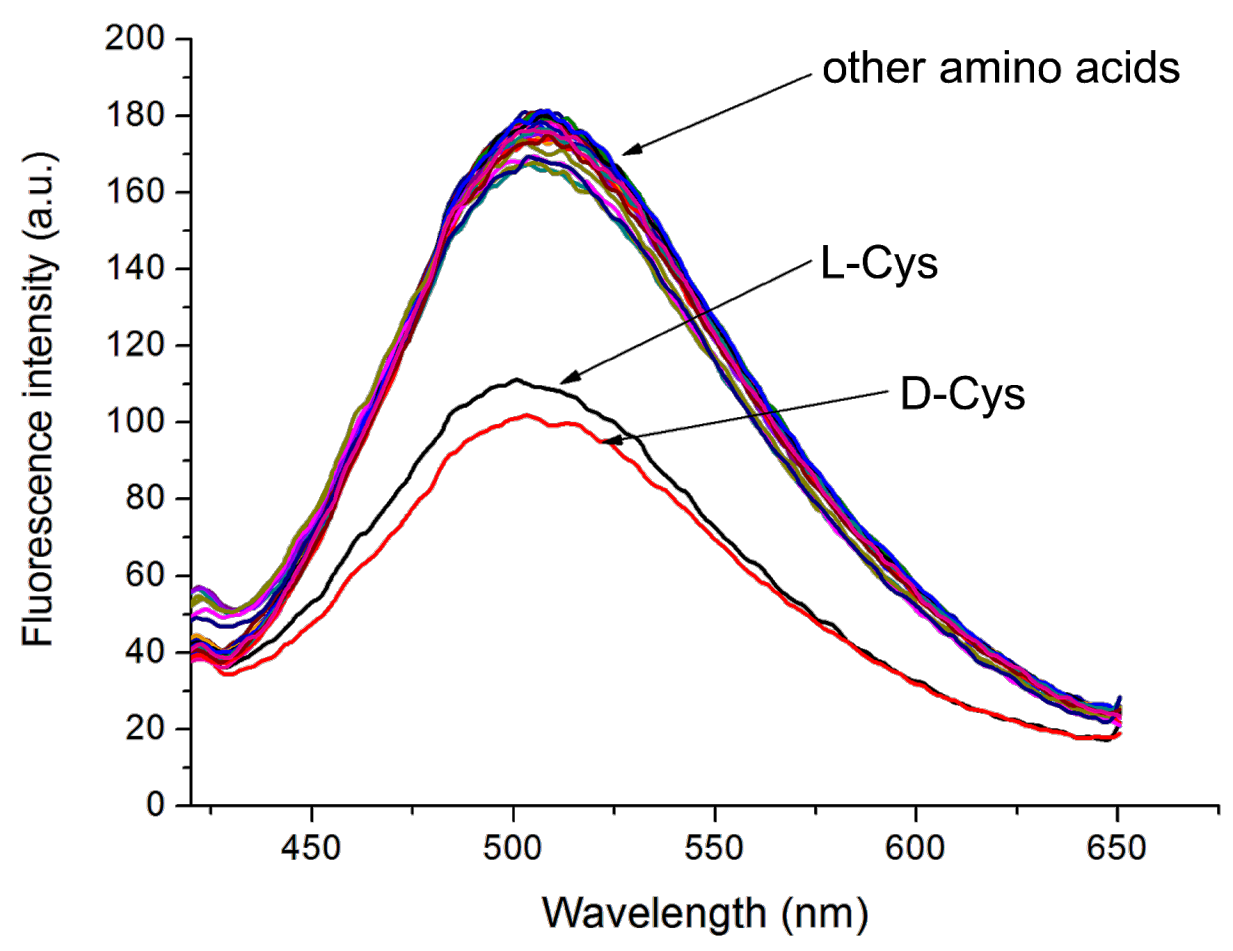
Fluorescence spectra of (10 µm) Cd^2+^–**ACAQ** upon the titration of various amino acids in buffer solution (10 mM, HEPES, 1% ACN, pH 7.4) at 298 K (excitation at 350 nm). Other amino acids include: GSH; L-Met; DL-Hcy; *N*-Ac-L-Cys; MPA; L-Asp; D-Asp; D-Glu; L-Glu; D-Lys; L-Lys; D-Ser; L-Ser; D-Tyr; L-Tyr; D-Val; L-Val; D-Phe; L-Phe; D-His; L-His.

As cadmium is well-known for its strong affinity to sulfur-containing compounds, we envisioned that the Cd^2+^-bound **ACAQ** complex would demonstrate chemoselective binding toward amino acids. Among 22 amino acids, a tripeptide, cysteine derivative and β-mercaptopropionic acid (MPA), only Cys demonstrated strong interaction with the probe (Figure 6). As evidenced by the relative fluorescence quenching ability of the thiols, it is noteworthy that only marginal bindings of the probe to glutathione (GSH) and DL-Hcy were found. The corresponding fluorescence response of each of the analytes to the probe can be found in Figure 7. In the control experiments, when 20 equivalents of the amino acids were added to **ACAQ** solutions under the same measuring conditions, the weak emission peak of the native **ACAQ** centred at 405 nm experienced an intensity change of less than 5%. Thus, the role of Cd^2+^ in the function of the sensory probe is evident and indispensible.

**Figure 8:**
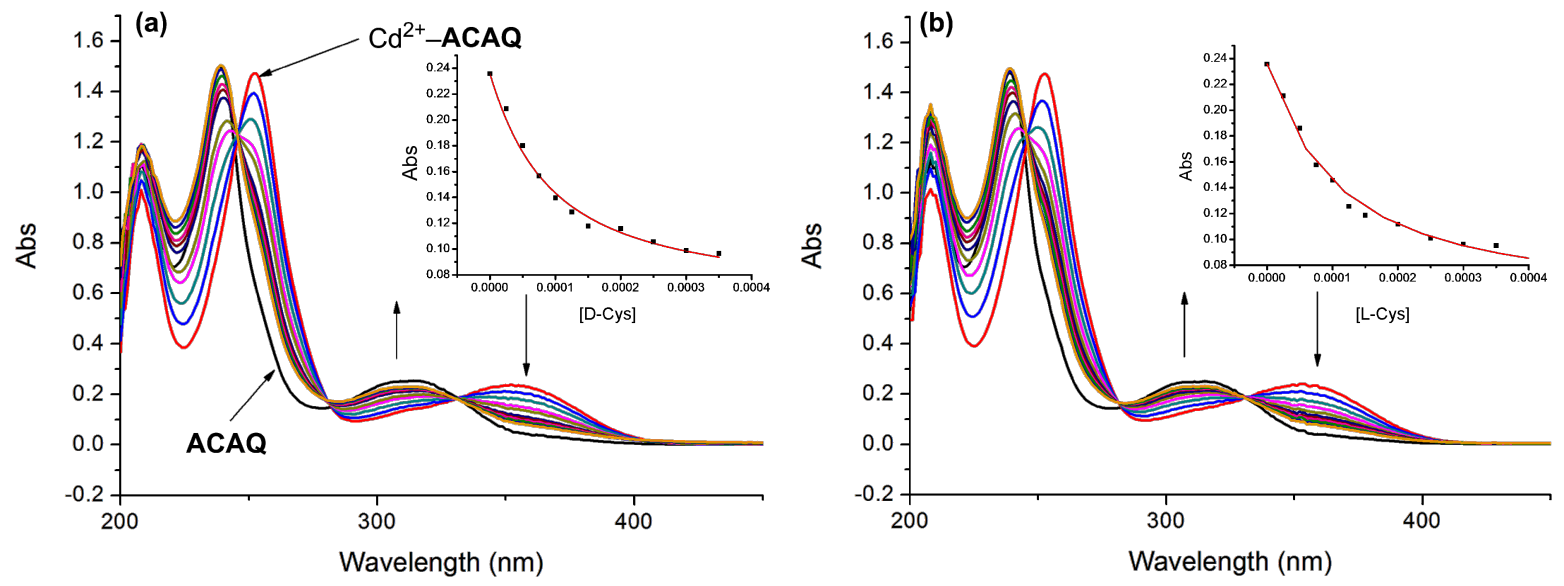
UV spectra of Cd^2+^–**ACAQ** (25 µm) upon the titration of (a) D-Cys, (b) L-Cys (0–12 equiv) in buffer solution (10 mM , HEPES, 50% ACN, pH 7.4 ) at 298 K. The inset shows the titration data points and the nonlinear least-squares fitting curve.

Furthermore, the binding interaction between the probe and the antipodal cysteines was investigated by means of UV titrations. In corroboration with the fluorescence study, a clear isosbestic point at around 330 nm was apparent when increasing concentrations of Cys were introduced into the buffered 1:1 ACN/HEPES solution of Cd^2+^–**ACAQ** (25 μM). The higher concentration of the sensor compared to that used in the fluorescence study led to the deterioration of its chiral recognizing ability for cysteines, to give a lower value of 1.22 (K_D_/K_L_) (Figure 8).

Considering the fact that Cys is a trifunctional amino acid, it is interesting to unravel its binding mode with the cadmium-centred fluorescent chemosensor **ACAQ**. To shed light on the binding mechanism of Cys with the probe, seven other sulfur-containing molecules were chosen as control compounds (Table 3). The extent of fluorescence quenching of the sensory ensemble at 500 nm triggered by the addition of 20 equivalents of each of the guest molecules was determined, and the results are shown in Table 3. Upon careful investigation of the relative quenching efficiency of the guest molecules on the sensor, it becomes apparent that the presence of free amine and thiol groups is essential for the effective interaction with the cadmium metal centre of the sensor. Interestingly, Hcy bearing a β-mercaptoamino moiety is far less effective at binding with the cadmium of the probe in comparison with the α-mercaptoamino group present in Cys. When the thiol group in Hcy is methylated, as in methionine, its power to bind to the sensor is completely lost. As none of the bifunctional α-amino acids caused any fluorescence quenching of the probe, the presence of β-mercaptoamino moiety in cysteine may confer a synergistic binding affinity of its carboxyl group to Cd^2+^–**ACAQ**. Such a proposition was supported by the observation that by converting the carboxyl group of cysteine into its methyl ester, the quenching ability of the derivative toward the probe was greatly reduced.

**Table 3:** The quenching factor of Cd^2+^–**ACAQ** with various thiols.

guest	quenching factor	guest	quenching factor

D-Cys	0.43	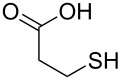	0.99
L-Cys	4.57		
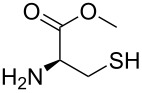	0.72	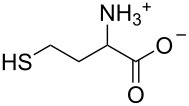	0.85
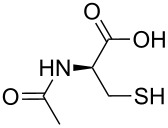	0.93	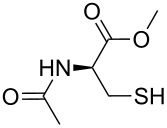	0.98
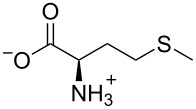	0.99	GSH	0.83

**Figure 9:**
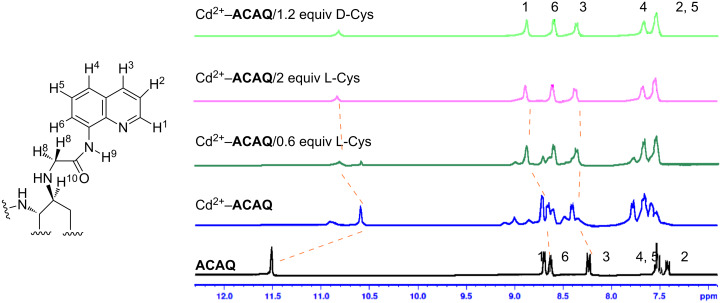
Partial ^1^H NMR spectra (400 MHz) of **ACAQ** (5 mM) before and after the addition of Cd^2+^ and then incremental addition of cysteine in DMSO-*d*_6_.

**Figure 10:**
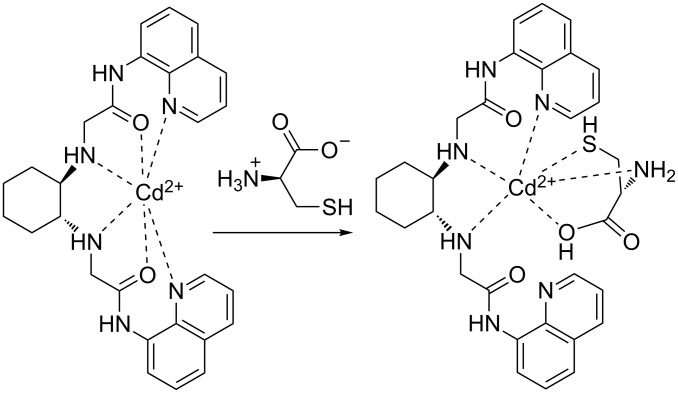
Proposed binding model of Cd^2+^–**ACAQ** with cysteine.

To evaluate the binding mode of the Cd^2+^–**ACAQ** complex with Cys, detailed ^1^H NMR titrations were carried out. Upon gradual addition of Cys to the DMSO-*d*_6_ solution of the ensemble, the amide proton resonating at δ 10.60 experienced a downfield shift to δ 10.75, implying a weakening of the interaction between the carboxamido oxygen and cadmium, presumably due to the ligating effect of Cys on cadmium. While Cys exerts its coordinating influence on cadmium, the coordination of the other nitrogen chelating sites of **ACAQ** with cadmium is reduced. As a result of the “demetalation” from **ACAQ,** triggered by the addition of Cys, all protons in the vicinity of these ligating groups underwent resonance shifts to different extents. For instance, as shown in Figure 9, a considerable downfield/upfield shift of H1, H2 and H3 was observed. It should be noted that a larger downfield shift of H1 in **ACAQ** was induced by the addition of D-Cys in comparison with that with L-Cys (Δδ = 0.08 vs 0.05). With the support of all the mentioned spectral evidence, the binding model of the probe is proposed in Figure 10.

**Figure 11:**
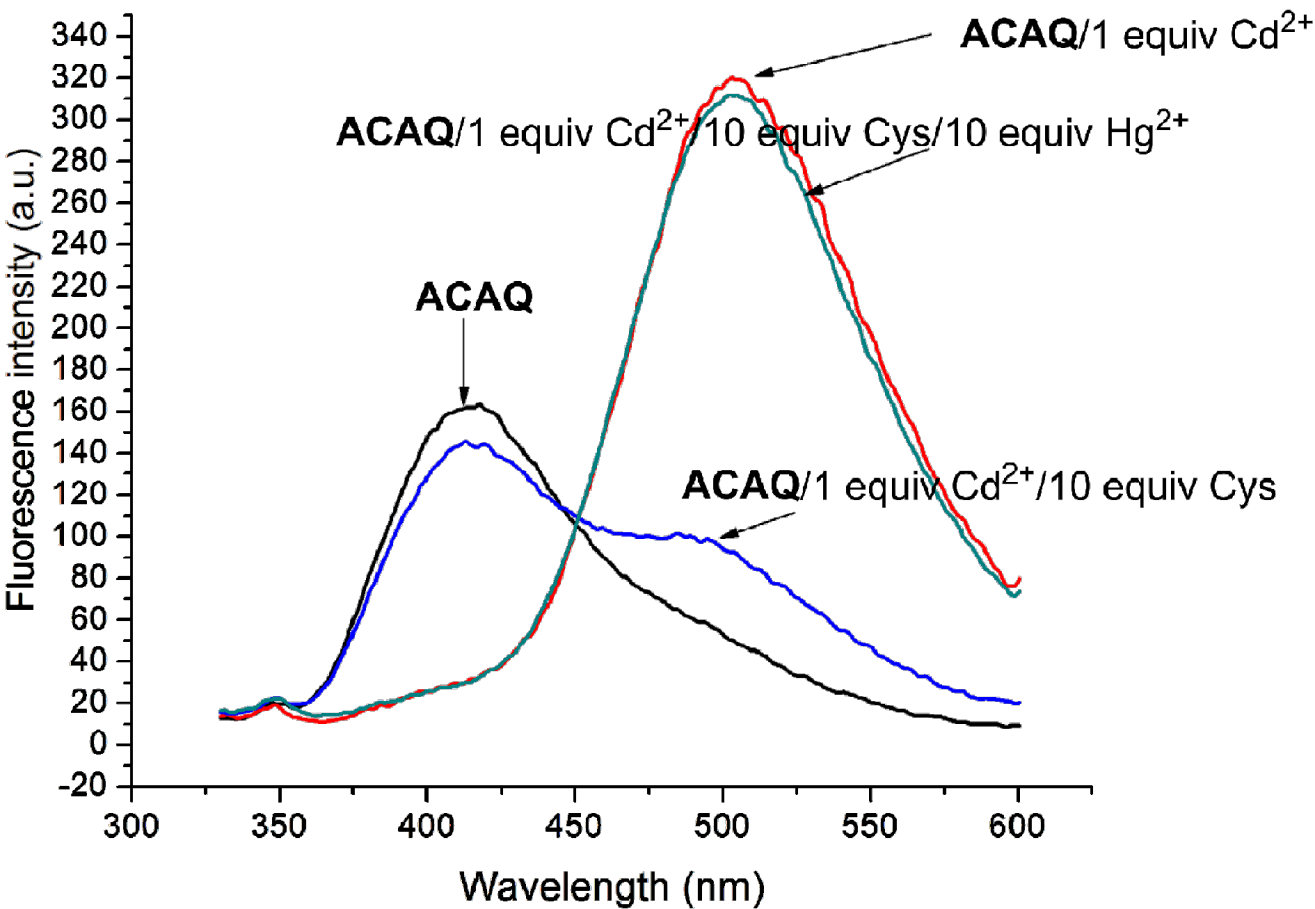
Reversibility study. Emission spectra of Cd^2+^–**ACAQ** complex (5 μM) with 10 equiv L-Cys in buffer solution (10 mM, HEPES, 80% ACN, pH 7.4) at 298 K, then 10 equiv of Hg^2+^. Excitation wavelength was 315 nm.

To rule out the possibility that the observed fluorescence ratiometric change is due to chemical reaction (i.e. chemodosimeter), the reversible binding of Cys and the probe must be established. As shown in Figure 11, the fluorescence spectrum of Cd^2+^–**ACAQ** can be fully recovered when 10 equivalents of Hg^2+^, which is a scavenger of Cys, was introduced.

## Conclusion

In summary, a simple, water-soluble, “chemosensing-ensemble” probe for chemo- and enantioselective detection of D-Cys was described. Among all amino acids that were investigated, only Cys and Hcy were responsive to the probe. Significantly, the probe not only demonstrated its outstanding discriminative power on Cys over Hcy and GSH in 99% aqueous buffered solution, but it also exhibited modest enantioselectivity on Cys for the first time.

## Experimental

Proton NMR spectra were recorded on a Bruker Avance-III 400 spectrometer at 400 MHz in DMSO-*d*_6_. Fluorescence emission spectra and UV–vis spectra were collected on a PE LS50B and a Cary UV-100 spectrometer, respectively. All inorganic reagents and amino acid derivatives were of analytical reagent grade and were obtained from Aldrich or Sigma. **ACAQ** was synthesized according to our method [16]. The stock solution of 1.0 × 10^−3^ M **ACAQ** was obtained by dissolution of the compound in ACN. The stock solutions of 5.0 × 10^−3^ M amino acids were prepared by dissolution of the material in water. Working solutions were obtained by series dilution with HEPES, pH 7.4 solution.

Association constants (1:1) of Cd^2+^–ACAQ with anions were calculated by nonlinear least-squares curve ﬁtting with the following equation in Origin 7.5:


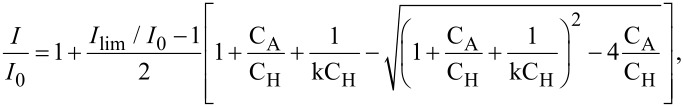


where *I*_0_ is the fluorescence intensity of the host without anions, *I*_lim_ is the limit of the fluorescence intensity upon addition of excessive anions, C_A_ is the concentration of anions added, and C_H_ is the concentration of the host molecule. The *I* was replaced with A, the absorption of UV–vis, when the UV–vis spectra were used.
